# Potential Role of the Renal Arterial Resistance Index in the Differential Diagnosis of Diabetic Kidney Disease

**DOI:** 10.3389/fendo.2021.731187

**Published:** 2022-01-14

**Authors:** Haiyang Li, Yunzhu Shen, Zhikai Yu, Yinghui Huang, Ting He, Tangli Xiao, Yan Li, Jiachuan Xiong, Jinghong Zhao

**Affiliations:** Department of Nephrology, The Key Laboratory for the Prevention and Treatment of Chronic Kidney Disease of Chongqing, Chongqing Clinical Research Center of Kidney and Urology Diseases, Xinqiao Hospital, Army Medical University (Third Military Medical University), Chongqing, China

**Keywords:** diabetic kidney disease, non-diabetic kidney disease, resistance index, differential diagnosis, type 2 diabetes mellitus

## Abstract

**Aims:**

To investigate the potential role of renal arterial resistance index (RI) in the differential diagnosis between diabetic kidney disease (DKD) and non-diabetic kidney disease (NDKD) and establish a better-quantified differential diagnostic model.

**Materials and Methods:**

We consecutively reviewed 469 type 2 diabetes patients who underwent renal biopsy in our center. According to the renal biopsy results, eligible patients were classified into the DKD group and the NDKD group. The diagnostic significance of RI was evaluated by receiver operating characteristic (ROC) curve analysis. Logistic regression analysis was used to search for independent risk factors associated with DKD. Then a novel diagnostic model was established using multivariate logistic regression analysis.

**Results:**

A total of 332 DKD and 137 NDKD patients were enrolled for analysis. RI was significantly higher in the DKD group compared with those in the NDKD group (0.70 vs. 0.63, *p*< 0.001). The optimum cutoff value of RI for predicting DKD was 0.66 with sensitivity (69.2%) and specificity (80.9%). Diabetic retinopathy, diabetes duration ≥ 60 months, HbA1c ≥ 7.0(%), RI ≥ 0.66, and body mass index showed statistical significance in the multivariate logistic regression analysis. Then, we constructed a new diagnostic model based on these results. And the validation tests indicated that the new model had good sensitivity (81.5%) and specificity (78.6%).

**Conclusions:**

RI has a potential role in discriminating DKD from NDKD. The RI-based predicting model can be helpful for differential diagnosis of DKD and NDKD.

## Introduction

Diabetes mellitus (DM) is a global public health challenge affecting over 463 million adults, according to the report of the International Diabetes Federation in 2019 (1). In China, it is estimated that approximately 11.2% of the population has DM (129.8 million people) (2). Type 2 diabetes mellitus (T2DM) combined with renal impairment is correlated with increased cardiovascular mortality and all-cause mortality (3). Diabetic kidney disease (DKD) is now one of the most frequent and severe complications of diabetes and continues to be the principal cause of end-stage kidney disease (ESKD) worldwide (4, 5). However, non-diabetic kidney disease (NDKD) occurs in T2DM patients as well (6). The prevalence of NDKD in T2DM varied widely from 36.8% to 82.9% (7–12). The therapy and prognosis of NDKD are pretty different from DKD (13, 14). It is believed that the renal outcomes of patients with DKD are relatively worse compared with their counterparts with biopsy-proven NDKD because the pathological changes of DKD are deemed difficult to reverse (15, 16). Therefore, it is critical to distinguish between DKD and NDKD in diabetes with renal impairment in clinical practice.

Currently, the renal pathological diagnosis is the gold standard to discriminate DKD from NDKD. However, the kidney biopsy is an invasive procedure that is impracticable in patients with contraindications, such as pyknotic kidney, bleeding tendency, solitary kidney, uncontrolled hypertension, or severe anemia. Moreover, a renal biopsy could not be routinely performed in some primary hospitals. Thus, the diagnosis and appropriate treatment were usually based on clinical indicators, such as diabetes duration, hematuria, diabetic retinopathy (DR), glycated hemoglobin (HbA1c), and other indices (17–22). However, those markers are not entirely accurate. For instance, lack of DR contributes to the diagnosis of NDKD but does not rule out DKD (23). In recent years, some studies have used some new markers and diagnostic models for the clinical differentiation between DKD and NDKD, such as dysmorphic erythrocytes and urinary neutrophil gelatinase-associated lipocalin (22, 24). However, these markers or risk model is not perfect and is still not good enough to meet clinical requirements. Therefore, it is necessary to find a new precise and sensitive non-invasive marker for clinical differentiation of DKD from NDKD.

The Renal atrial resistance index (RI), measured by doppler ultrasound, is a low-cost and non-invasive tool in detecting kidney diseases; it has been extensively used to evaluate renal blood flow as a semi-quantitative parameter. Previous studies suggested that RI is correlated with severe interstitial fibrosis and the progression of chronic kidney disease (CKD) (25–27). In addition, a few studies have noticed that RI in patients with DKD is significantly higher when compared with non-diabetic controls, which might be helpful for the identification and prediction of DKD (28, 29). However, the potential role of RI in the clinical differentiation of DKD from NDKD and the optimal cutoff value remains unclear. Thus, the present study was conducted to investigate the potential role of RI in the differential diagnosis between DKD and NDKD and establish a better-quantified differential diagnostic model.

## Materials and Methods

### Study Subjects

**Figure 1:**
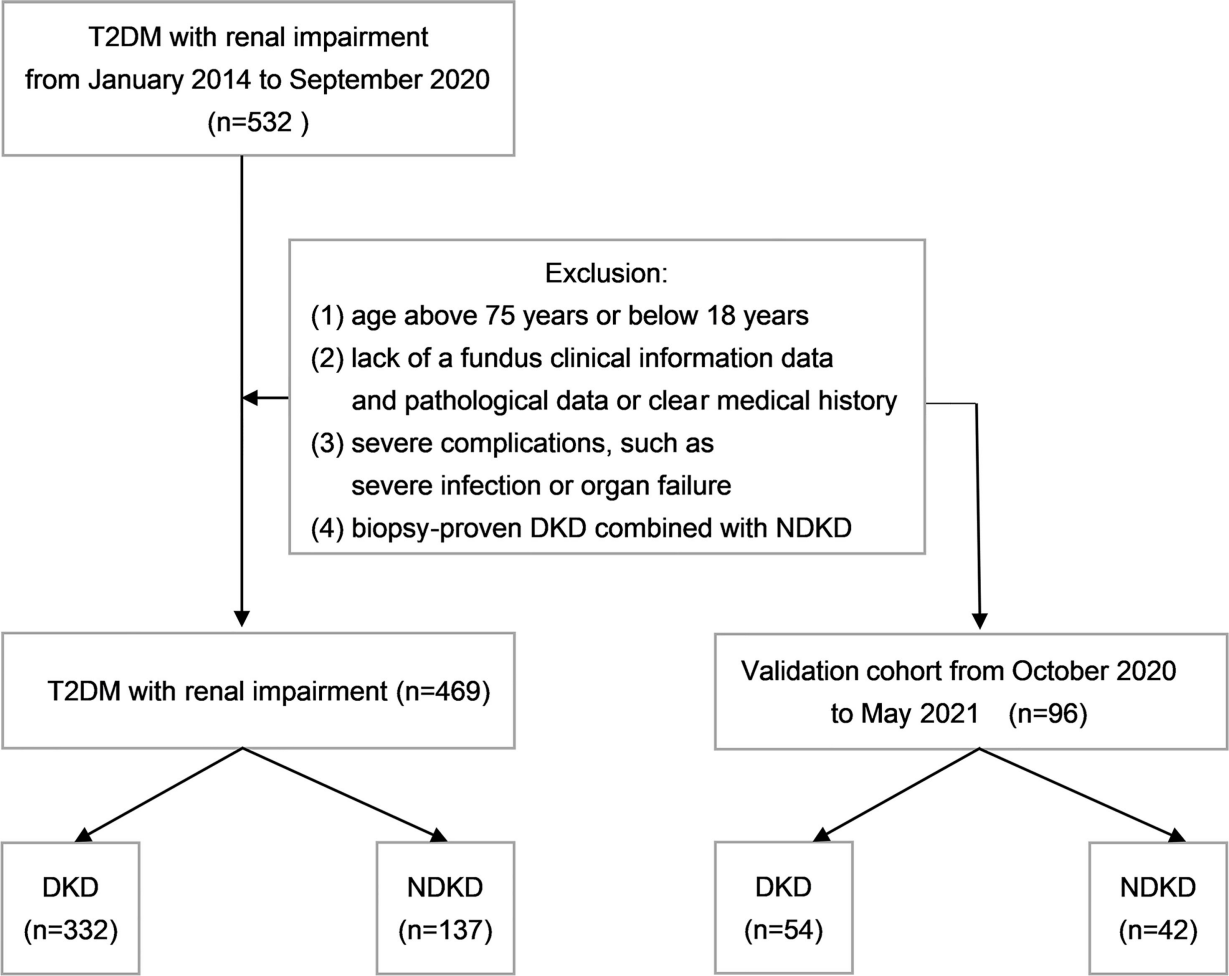
Flowchart showing the procedure for the selection of study participants. T2DM, type 2 diabetes mellitus; DKD, diabetic kidney disease; NDKD, non-diabetic kidney disease.

A total of 469 T2DM patients with renal impairment from the department of nephrology at Xinqiao Hospital, Army Medical University, from January 2014 to September 2020 were retrospectively analyzed (**Figure 1**). All patients had received echo-color-Doppler examination of renal vessels, systematic screening for diabetic retinopathy, and renal biopsy. The diagnosis of T2DM met the criteria proposed by American Diabetes Association in 2019 (30). Eligible patients were divided into the DKD group and the NDKD group based on the kidney biopsy results. The inclusion criteria were: T2DM patients with renal impairment and received kidney biopsy; serum creatinine < 442 μmol/L. The exclusion criteria were: age above 75 years or below 18 years; lack of a fundus clinical information data and pathological data or clear medical history; severe complications, such as severe infection, heart failure, and hypertensive emergency; biopsy-proven DKD complicated by NDKD. All patients signed informed consent before kidney biopsy. The study was approved by the ethical committee of Xinqiao Hospital, and was in accordance with the principles of the Declaration of Helsinki.

### Renal Biopsy and Pathological Examination

The kidney biopsies were performed by an experienced renal pathologist, and every kidney biopsy tissue was investigated by electron microscopy, light microscopy, and immunofluorescence. The kidney biopsy indications were in accordance with the KDOQI Guideline (31). The pathological diagnostic criteria for DKD was: diffuse mesangial proliferation, diffuse capillary glomerulosclerosis, presence of Kimmelstiel–Wilson nodular lesions, glomerular basement membrane thickening, hyaline exudative lesions (32). NDKD usually has some unique features based on the guidelines previously reported (12).

### Clinical and Laboratory Information

The following data were collected at the time of kidney biopsy: age, sex, body mass index (BMI), systolic blood pressure, diastolic blood pressure, presence of hypertension and DR, medical history of DM, fasting blood glucose, HbA1c, hemoglobin, platelet, serum albumin, total cholesterol, triglyceride, serum creatinine, uric acid, blood urea nitrogen, 24-hour urine protein, hematuria, and RI value. Blood pressure was measured three times according to a standard protocol, and the average value was calculated. DR was confirmed by fundus photography. Ultrasonography was performed by a Philips IU22 Cart G Ultrasound System with C5-1(Made in United Kingdom). The estimated glomerular filtration rate (eGFR) was obtained using the chronic kidney disease epidemiology collaboration (CKD-EPI) equation (33).

### Statistical Analysis

Continuous variables were shown as average ± standard deviation or the median and interquartile depending on the data distribution, whereas enumeration data were described as percentages (%). The t-test was applied for normally distributed data, and the Mann-Whitney U test was applied for non-normally distributed data. The chi-squared test was applied for enumeration data. Receiver operating characteristic (ROC) curve analysis was used to explore the optimal cutoff point of the RI to predict DKD. Univariate and multivariate logistic regression analyses (stepwise forward) were performed to search the independent risk factors relating to the DKD diagnosis, with results shown as the odds ratio (OR) and 95% confidence interval (CI). The final significant risk factors were included in two differential diagnostic models (with or without RI). The equation is as follows: *PDKD* = *exp (α + β1x1+ β2x2+ β3x3+…+βnxn)/[1 + exp(α + β1x1+ β2x2+ β3x3+…+ βnxn)].* PDKD is the probability of DKD diagnosis, α is a constant, β is the estimator, and x is the clinical predictor. If PDKD ≥ 0.5, the patient should be considered as DKD, while the diagnosis should be NDKD if PDKD < 0.5. The Delong test and the calculation of the net reclassification improvement and the integrated discrimination improvement were performed by R language to analyze two models. Then, a better model was selected. Finally, a back-substitution and a validation test (by a validation cohort of 96 cases) were conducted to evaluate the new model. Correlations between RI and clinical indices were analyzed by the Pearson test. Statistical analyses were performed by SPSS (IBM SPSS Statistics 23.0) and R language. P < 0.05 was considered statistically significant. The number of patients required for the validation cohort is computed using software PASS 15.0.5.

## Results

### The Clinical Characteristics and Renal Pathological Features of the Included Patients

A total of 469 patients were divided into two groups based on kidney biopsy results, with 332 patients in the DKD group and 137 patients in the NDKD group. The general clinical information of the two groups was shown in **Table 1**. Compared with the NDKD group, patients in the DKD group had longer diabetes duration, higher incidence of DR, and higher levels of systolic blood pressure, fasting blood glucose, HbA1c, serum creatine, blood urea nitrogen and RI value, while lower levels of BMI, hemoglobin, triglyceride and eGFR. But no significant difference was noticed between the two groups regarding age, gender, diastolic blood pressure, platelets, serum albumin, total cholesterol, uric acid, urinary protein, or presence of hematuria, nephrotic syndrome, hypertension, cardiovascular disease. Moreover, renal pathological findings showed that membranous nephropathy (38 cases, 27.74%) and IgA nephropathy (38 cases, 27.74%) were the most common pathological type among the 137 NDKD patients, followed by mesangial proliferative glomerulonephritis (19 cases, 13.87%), hypertensive nephrosclerosis (16, 11.68%), and other types (**Supplementary Table 1**). The main pathological manifestations of the DKD group were advanced lesions, with 71.08% of the total were classified as class III (**Supplementary Table 2**).

**Table 1:** The general clinical characteristics of the included patients.

Parameter		All cases	NDKD	DKD	P value
		(n = 469)	(n = 137)	(n = 332)	
Age, (years)		51.53 ± 10.26	52.04 ± 11.05	51.32 ± 9.93	0.489
Gender, (male, %)		299 (63.75)	82 (59.85)	217 (65.36)	0.259
BMI, (kg/m^2^)		25.24 ± 3.48	26.51 ± 4.01	24.80 ± 3.42	<0.001
SBP, (mm Hg)		142.73 ± 22.62	136.62 ± 21.42	145.26 ± 22.69	<0.001
DBP, (mm Hg)		84.43 ± 12.16	84.32 ± 12.58	84.47 ± 12.00	0.905
Duration of diabetes, (months)		76.03 ± 62.41	28.10 ± 30.59	94.66 ± 61.71	<0.001
Duration of diabetes ≥ 60 months (%)		240 (51.17)	23 (16.79)	217 (65.35)	<0.001
HbA1c		7.82 ± 2.14	7.32 ± 1.90	8.01 ± 2.19	0.004
HbA1c ≥ 7, (%)		216 (46.06)	45 (32.85)	171 (51.51)	<0.001
FBG, (mmol/L)		7.34 ± 3.33	6.76 ± 2.92	7.58 ± 3.46	0.015
Hemoglobin, (g/L)		117.51 ± 24.93	127.02 ± 22.02	113.59 ± 25.03	<0.001
PLT, (10^9/L)		205.76 ± 79.36	206.44 ± 80.91	205.48 ± 78.84	0.906
Serum albumin, (g/L)		35.05 ± 9.43	34.20 ± 10.81	35.40 ± 8.79	0.215
TC, (mmol/L)		5.47 ± 1.98	5.65 ± 2.45	5.40 ± 1.76	0.217
TG, (mmol/L)		2.21 ± 1.86	2.57 ± 2.19	2.07 ± 1.69	0.008
Uric acid, (μmol/L)		378.59 ± 96.49	372.42 ± 108.05	381.04 ± 91.57	0.388
Scr, (μmol/L)		127.32 ± 76.47	102.41 ± 60.89	137.52 ± 79.87	<0.001
BUN, (mmol/L)		7.86 ± 3.67	6.89 ± 3.39	8.26 ± 3.71	<0.001
eGFR, (ml/min/1.73m^2^)		68.08 ± 35.44	82.67 ± 34.37	61.32 ± 34.23	<0.001
Urinary protein, (g/24 h)		3.41 ± 3.71	3.49 ± 4.39	3.38 ± 3.41	0.778
Hematuria (%)		320 (68.23)	96 (70.07)	224 (67.47)	0.372
RI		0.68 ± 0.07	0.63 ± 0.05	0.70 ± 0.07	<0.001
Clinical comorbidities					
Nephrotic syndrome (%)		109 (23.24)	32 (23.36)	77 (23.19)	0.969
Hypertension (%)		325 (69.29)	90 (65.69)	235 (70.78)	0.282
Cardiovascular disease (%)		180 (38.38)	52 (37.96)	128 (38.55)	0.799
DR (%)		227 (48.40)	12 (8.76)	215 (64.76)	<0.001

BMI, body mass index; SBP, systolic blood pressure; DBP, diastolic blood pressure; HbA1c, glycosylated hemoglobin; FBG fasting blood glucose; PLT, platelet; TC total cholesterol; TG triglyceride; Scr serum creatine; BUN blood urea nitrogen; RI, resistance index; DR, diabetic retinopathy. Data were presented as the mean ± standard, the median with range or counts and percentages. A two-tailed P < 0.05 was considered statistically significant.

### Differential Diagnosis Performance of RI in Diabetic Patients With Renal Impairment

**Figure 2:**
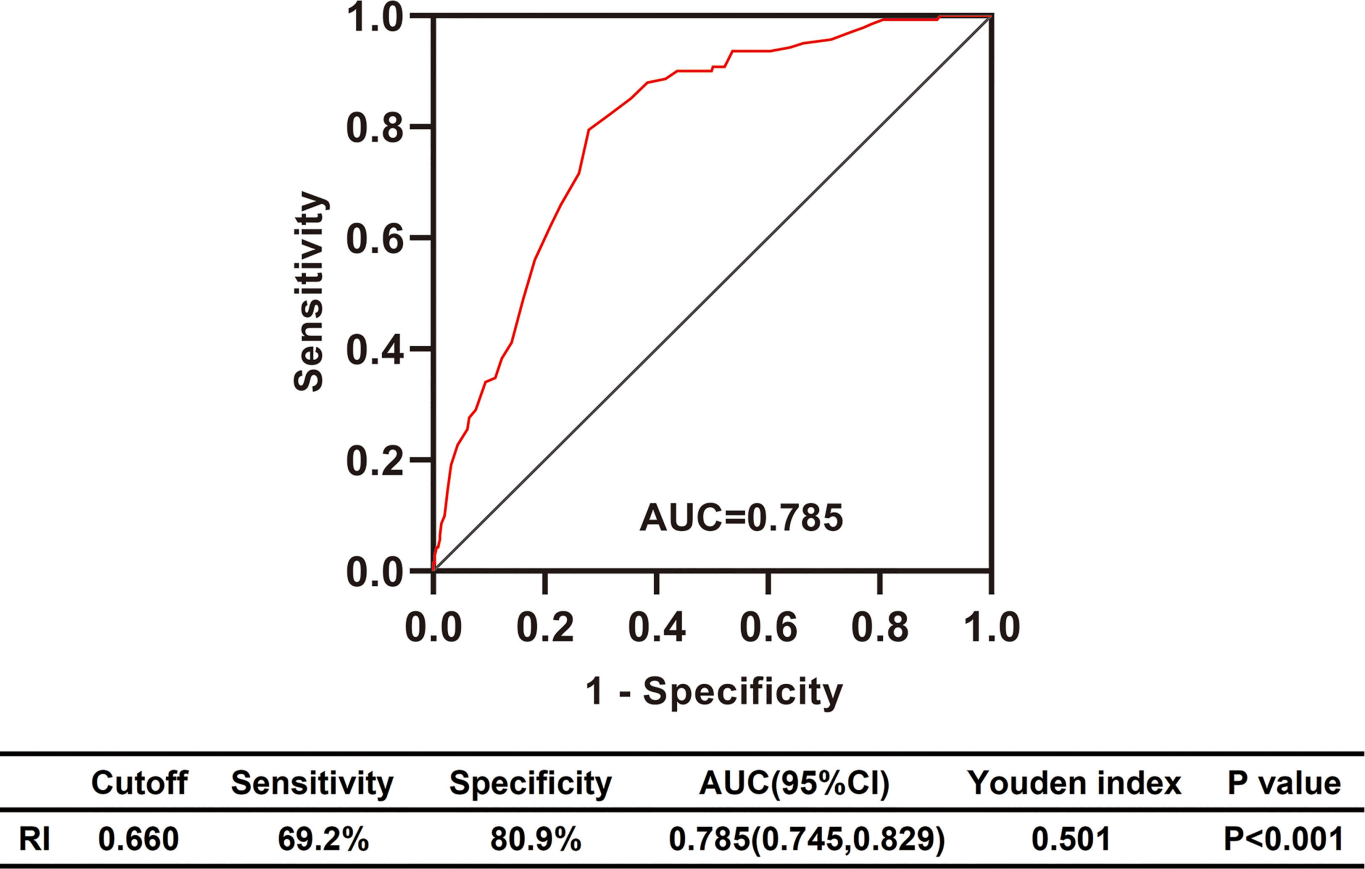
Differential diagnosis performance of RI in type 2 diabetic patients with renal impairment evaluated by ROC curve. RI, resistance index; AUC, the area under ROC curve; CI, confidence interval; ROC, receiver operating characteristic.

To explore the clinical value of RI in DKD and NDKD, we compared the RI value in the two groups. Results showed patients in the DKD group had a significantly higher RI value compared with those in the NDKD group (0.70 *vs.* 0.63, *p*<0.001, **Table 1**). Then, we performed a ROC curve to determine the cutoff point of RI for predicting DKD. The area under the curve (AUC) of RI was 0.785. The best cutoff point of RI was 0.66, with 69.2% sensitivity and 80.9% specificity, as calculated by obtaining the best Youden index (**Figure 2**). Then, we assigned a value of 1 to RI ≥ 0.66 and converted it into a binary variable. Considering the value of RI in the differential diagnosis of two groups, the correlation analysis between RI and other clinical information was performed. There were 256 (54.6%) patients with RI ≥ 0.66 and 213 (45.4%) patients with RI < 0.66, respectively. Compared with patients with RI < 0.66, patients with RI ≥ 0.66 had higher age, systolic blood pressure, duration of diabetes, serum creatinine, blood urea nitrogen, the proportion of hypertension, diabetic retinopathy, and nephrotic syndrome, while a lower level of BMI, serum albumin, hemoglobin, TG, and eGFR (**Table 2**). Further analyses was performed by linear regression, RI levels were positively correlated with age (r=0.245; *p*<0.001), duration of diabetes (r=0.341; *p*<0.001), systolic blood pressure (r=0.274; *p*<0.001); serum creatinine (r =0. 335; *p*<0.001); blood urea nitrogen (r=0.037; *p*<0.001) and total urinary protein, g/24 h (r=0.141; *p*=0.003), whereas it was inversely correlated with BMI (r=−0.143; *p*=0.002), hemoglobin (r=−0.424; *p*<0.001), triglyceride (r=−0.145; *p*=0.002), and serum albumin (r=−0.163; *p*<0.001) in diabetic patients with renal impairment (**Supplementary Table 3**).

**Table 2:** Comparison of clinical findings in T2DM with renal impairment according to renal resistance index.

Parameters	R I< 0.66 (n = 213)	RI ≥ 0.66 (n = 256)	P value
Age, (years)	49.20 ± 10.47	53.37 ± 9.64	<0.001
Gender, (male, %)	132(61.97)	166 (64.84)	0.526
BMI, (kg/m^2^)	25.67 ± 3.64	24.87 ± 3.31	0.013
SBP, (mm Hg)	136.73 ± 20.47	147.62 ± 23.17	<0.001
DBP, (mm Hg)	84.92 ± 11.53	83.96± 12.69	0.398
Duration of diabetes, (months)	51.46 ± 55.12	95.69 ± 61.01	<0.001
Duration of diabetes ≥ 60, months (%)	70 (32.86)	169(66.01)	<0.001
HbA1c, (%)	7.70 ± 1.88	7.94 ± 2.33	0.284
HbA1c ≥ 7, (%)	97(45.54)	118 (46.09)	0.832
FBG, (mmol/L)	7.17 ± 3.08	7.48 ± 3.54	0.313
Hemoglobin, (g/L)	126.00 ± 23.53	110.60 ± 23.95	<0.001
PLT, (10^9/L)	211.45± 82.92	200.98 ± 76.00	0.157
Serum albumin, (g/L)	36.13 ± 10.45	34.26 ± 8.37	0.034
TG, (mmol/L)	2.44 ± 1.97	2.03 ± 1.75	0.018
TC, (mmol/L)	5.47 ± 2.24	5.48 ± 1.75	0.920`
Scr, (μmol/L)	106.47± 60.54	144.76 ± 83.96	<0.001
eGFR, (ml/min/1.73m^2^)	79.03 ± 35.72	59.22 ± 32.71	<0.001
BUN, (mmol/L)	7.01 ± 3.21	8.56 ± 3.90	<0.001
Uric acid, (μmol/L)	375.98 ± 103.04	381.33 ± 91.05	0.555
Urinary protein, (g/24 h)	3.03 ± 3.96	3.73 ± 3.47	0.054
Hematuria (%)	134 (62.91)	184 (71.88)	0.056
Cardiovascular disease (%)	78 (36.61)	101 (39.45)	0.479
Hypertension (%)	121 (56.81)	191 (74.61)	0.003
DR (%)	63 (29.58)	163 (63.67)	<0.001
Nephrotic syndrome (%)	40 (18.78)	69 (26.95)	0.037

BMI, body mass index; SBP, systolic blood pressure; DBP, diastolic blood pressure; HbA1c, glycosylated hemoglobin; FBG fasting blood glucose; PLT, platelet; TG triglyceride; TC total cholesterol; Scr serum creatine; BUN blood urea nitrogen; DR, diabetic retinopathy. Data were presented as the mean ± standard, the median with range or counts and percentages. A two-tailed p < .0.05 was considered statistically significant.

### Screening for DKD Diagnosis-Related Factors

Previous studies found that the duration of diabetes ≤ 60 months was an independent risk factor for NDKD (11). Many researches indicated that intensive blood glucose control (HbA1c 6.5–7.0%) could reduce the risk of DKD. HbA1c <7.0 was considered as a common indicator of clinical blood glucose control (34). Therefore, we assigned a value of 1 to the duration of diabetes ≥ 60 months and HbA1c ≥ 7% respectively. Then those indicators were converted into binary variables. Univariate regression analysis indicated that duration of diabetes ≥ 60 months, BMI, systolic blood pressure, DR, fasting blood glucose, HbA1c ≥ 7%, hemoglobin, triglyceride, serum creatinine, blood urea nitrogen, and RI ≥ 0.66 were related to the diagnosis of DKD. After adjusting for the factors mentioned above using multivariate logistic regression analysis, RI ≥ 0.66 was still an independent risk factor for the DKD diagnosis, as well as the duration of diabetes ≥ 60 months, BMI, DR, and HbA1c ≥ 7% (**Table 3**).

**Table 3:** Predictors of DKD in T2DM with renal impairment.

Parameters	Univariate		Multivariate	
	OR (95%CI)	P value	OR (95%CI)	P value
Duration of diabetes ≥ 60 months (yes/no)	9.34 (5.61,15.56)	0.000	5.29 (2.63,10.59)	<0.001
BMI, (kg/m^2^)	0.89 (0.84,0.94)	0.000	0.90 (0.82,1.00)	0.045
SBP, (mmHg)	1.02 (1.01,1.03)	0.001		
DR (yes/no)	19.14 (10.16,36.07)	0.000	14.80 (6.40,34.26)	<0.001
HbA1c ≥ 7% (yes/no)	2.20 (1.40,3.45)	0.001	2.19 (1.14,4.21)	0.019
FBG, (mmol/L)	1.09 (1.02,1.17)	0.016		
Hemoglobin, (g/L)	0.98 (0.97,0.99)	0.000		
TG, (mmol/L)	0.87 (0.78,0.97)	0.014		
Scr, (μmol/L	1.01 (1.01,1.01)	0.000		
BUN, (mmol/L)	1.13 (1.06,1.21)	0.000		
RI ≥ 0.66 (yes/no)	9.50 (5.84,15.46)	0.000	5.19 (2.59,10.38)	<0.001

BMI, body mass index; SBP, systolic blood pressure; DR, Diabetic retinopathy; HbA1c, glycosylated hemoglobin; FBG, fasting blood glucose; TG, triglyceride; Scr, serum creatine; BUN, blood urea nitrogen; RI, resistance index; CI, confidence interval.

### Establishment and Validation of the New Differential Diagnostic Model

**Figure 3:**
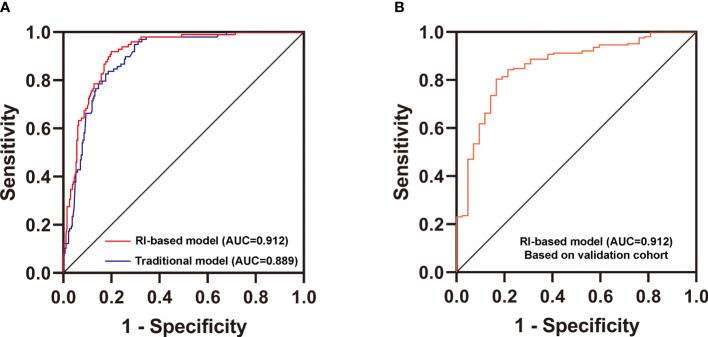
Validation of the new differential diagnostic model. **(A)** Comparison of the area under the curve (AUC) of the RI-based model and the traditional model. DeLong’s test was applied by R language to compare the AUC of two models (with or without RI). Z = 2.5964, P value = 0.00942. **(B)** Receiver operating characteristics (ROC) curve for the discriminative effect of the RI-based model in the Validation cohort. AUC, the area under the curve =0.857.

Then, we used two multivariate logistic regression analyses to establish two differential diagnostic models (with or without RI value) to explore the RI value for the clinical differentiation between DKD and NDKD (**Table 4**). The traditional model was built by four independent risk factors other than RI, and the RI-based model was built by five independent risk factors, including RI≥ 0.66. The detailed equation of the two models is shown in **Supplementary Table 4**. The area under ROC curve of the traditional model was 0.889. After adding RI, the area under the ROC curve of the RI-based model increased to 0.912 (**Figure 3A**). The statistical significance of two ROC AUC by DeLong’s test (Z = 2.5964, P value = 0.00942), the net reclassification improvement (NRI = 0.1837, P value = 0.00278), and the integrated discrimination improvement (IDI = 0.0572, P value = 0.00013) implied that the RI-based model has improved the efficiency of differential diagnosis. The sample size of the validation cohort was computed by software PASS based on the AUC of the traditional model (AUC=0.912). The results showed that a random sample of 51 subjects from the positive population and 40 from the negative population produced a two-sided 95.0% confidence interval with a width of 0.120. Then, we recruited another 96 patients (from October 2020 to May 2021) that met the same inclusion criteria to validate the RI-based model’s efficacy further. The Validation cohort test demonstrated that the RI-based model had a sensitivity of 81.5% and a specificity of 78.6% (**Table 5**). The ROC curve (AUC=0.857) based on the validation cohort indicated that the RI-based model had good diagnostic efficiency (**Figure 3B**).

**Table 4:** Development of two differential diagnostic mode.

Parameters	The RI-Based Model				The Traditional Model			
	OR (95%CI)	B	SE	P value	OR (95%CI)	β	SE	P value
Dm (0/1)	5.320 (2.653,10.66)	1.671	0.35	0.000	6.984 (3.617,13.483)	1.944	0.336	0.0000
BMI	0.904 (0.819,0.998)	-0.101	0.050	0.046	0.887 (0.809,0.973)	-0.120	0.047	0.011
DR (0/1)	14.921 (6.445,34.546)	2.703	0.428	0.000	15.790 (7.077,35.227)	2.759	0.409	0.000
Gh (0/1)	2.214 (1.151,4.258)	0.795	0.334	0.017	1.799 (0.978,3.311)	0.587	0.311	0.009
RI (0/1)	5.239 (2.618,10.483)	1.656	0.000	0.000				
Constant	2.655	0.976	1.318	0.459	8.137	2.096	1.204	0.082

OR, odds ratio; SE, standard error; Dm, diabetes duration ≥ 60 months (1 yes, 0 no); BMI, body mass index; DR, diabetic retinopathy (1 yes, 0 no); Gh, HbA1c ≥ 7.0% (1 yes, 0 no); RI, Resistance index ≥ 0.66 (1 yes, 0 no).

**Table 5:** Predictive value of the Back-substitution and Validation cohort test.

	Back-Substitution Test			Validation Cohort Test		
	DKD	NDKD	Total	DKD	NDKD	Total
Diagnosed as DKD	300	45	345	44	9	53
Diagnosed as NDKD	32	92	124	10	33	43
Total	332	137	469	54	42	96
Sensitivity		90.4%			81.5%	
Specificity		67.2%			78.6%	
Positive predictive value		87.0%			83.0%	
Negative predictive value		74.2%			76.7%	
Total consistency		83.6%			80.2%	

DKD, diabetic kidney disease; NDKD, non-diabetic kidney disease.

## Discussion

For T2DM patients with renal impairment, it is of great significance to differentiate between DKD and NDKD to guide clinical diagnosis and treatment. Kidney biopsy is the ultimate method of differential diagnosis, but its clinical application is restricted by many contraindications (35, 36). Therefore, it is necessary to find a new precise and sensitive non-invasive indicator for clinical differentiation of DKD from NDKD. The present study found that the RI value, which reflects vascular disease (37), was remarkably higher in the DKD group in contrast to the NDKD group. Furthermore, the RI-based differential model has good sensitivity and specificity.

Doppler ultrasound as a non-invasive, low-cost method has been extensively used in the detection of reno-vascular diseases. RI is calculated by the ratio of the difference between peak systolic velocity (PSV) and end-diastolic velocity (EDV) divided by peak systolic velocity (PSV), obtained by the Doppler spectrum analysis from segmental or interlobar arteries. PSV mainly reflects the degree of renal vascular filling and blood supply, while the EDV reflects renal blood perfusion, and RI mainly reflects vascular bed resistance (38). There is no uniform standard for RI average value. The normal mean renal RI value reported in previous literature are listed in **Supplementary Table 5**. Generally, a value of 0.60 is considered a normal value for renal RI (39, 40). Current studies have found that RI can effectively assess the renal blood perfusion status, whether for renal damage caused by hypertension and diabetes (41–43) or for risk prediction and disease assessment of early acute renal injury induced by various diseases (44–47). In patients with CKD, RI ≥ 0.70 is an independent risk factor for the progression of renal failure (25, 48). And RI ≥ 0.80 was associated with a lower survival rate (49). Moreover, studies have also confirmed that the RI is higher in the newly diagnosed and untreated DKD patients than healthy controls (28, 29, 50), even before the onset of microalbuminuria, supporting the dynamic evaluation of renal RI as an early detector of renal vascular alterations in the presence of T2DM (51). A previous study has also demonstrated a higher value of RI in DKD compared with diabetic patients without kidney disease (52). Different from previous studies, our study focused on comparing the RI level between the DKD group and the NDKD group. We found a significantly higher RI value in the DKD group. RI ≥ 0.66 was proved to be an independent risk factor for the diagnoses of DKD in T2DM patients with renal impairment, which could improve the efficiency of differential diagnosis in identifying cases with a higher clinical suspicion for DKD. It is worth noting that the RI value is affected by age, race, region and many other factors so that the best cutoff value may be different in different studies. The cutoff value of RI in this study was derived from a small group of people in this specific region. A more appropriate cutoff value of RI needs further clinical research with a larger sample size in the future.

Glomerular hemodynamic changes (including hyperfiltration and hypoperfusion) are key pathological processes in the development of DKD, which could explain why RI value as an indicator of renal hemodynamics has a potential value in the differential diagnosis of DKD (53). The cause of glomerular hyperfiltration is currently believed to be caused by an increase in the trans-glomerular pressure gradient. The high glucose leads to the glycation of the basement of small blood vessels, particularly the efferent arteriole, making it thicken and stiffer and increasing the pressure within the glomerulus. Simultaneously, the afferent arteriole dilates, letting more blood flow into the glomeruli and increasing the pressure even further (54). With the deterioration of DKD, renal perfusion pressure and glomerular filtration rate continue to rise, resulting in glomerular capillary wall thickening, permeability enhancement, vascular lumen stenosis, glomerular capillary pressure increases. Those changes eventually lead to increased blood flow forward resistance and other renal artery hemodynamic disorders. Ultrasound showed that PSV and EDV decreased, especially EDV decreased and RI increased (53). Then, glomerular capillary basement membrane (GBM) thickening and mesangial matrix increased gradually, leading to glomerulosclerosis (55). Patients with NDKD often have less vascular involvement, such as membranous nephropathy mainly characterized by podocyte changes, and IgA nephropathy is primarily characterized by mesangial proliferation (56, 57). Although both DKD and NDKD patients suffered from renal hemodynamics changes, more significant vascular lesions and a higher RI value were detected in DKD patients.

Our research also found that DR and diabetic duration are powerful predictors of DKD, which is consistent with previous studies (11, 18). Generally, the diabetes duration in DKD patients is longer than that in NDKD patients. Diabetes history ≥ 5 years is an independent risk factor for DKD (58). In the present study, the diabetes duration is defined as the time from the diagnosis of diabetes to the time of renal biopsy. The diagnosis is usually delayed in patients with T2DM. It is hard to clarify their actual course of diabetes before diagnosis, so DKD cannot be ignored due to a short history of diabetes. Similarly, DR is related to DKD (59). CKD patients with DR are conducive to the diagnosis of DKD, in which proliferative retinopathy has higher specificity. DR often accompanies kidney damage in T1DM, but the incidence of DR in T2DM is only 40%-60%. DR cannot completely distinguish DKD from NDKD because some diabetic patients without DR also have biopsy-proven DKD. Therefore, we choose to combine previous classic predictive indicators and RI that reflect vascular disease to construct a diagnostic model, significantly improving diagnostic efficiency. In addition, RI obtained by ultrasound examinations can be easily performed in most medical institutions, making our model more practical and economical in clinical practice. Therefore, we propose to incorporate the RI measure in the examination of DM combined with renal impairment. Interestingly, our study found for the first time that lower BMI was an independent risk factor for DKD. BMI was significantly lower in the DKD group than the NDKD group, which may be due to a longer duration of diabetes in the DKD group. However, compared with other independent risk factors, the OR value of BMI was only 0.9. Further studies are needed to power it as an independent risk factor for DKD.

There are several limitations in the present research. First of all, this research was a retrospective study performed in a single center with limited data. Secondly, all validation cohort patients came from the department of nephrology of Xinqiao Hospital, lacking external validation. Thirdly, we only measure RI value at the time of kidney biopsy, not continuous testing; certain errors are unavoidable.

In conclusion, the RI value might serve as a novel potential indicator in the differential diagnosis of DKD. The RI-based differential diagnostic model has improved the accuracy and could be commonly used in clinical practice.

## Data Availability Statement

The original contributions presented in the study are included in the article/**Supplementary Material**. Further inquiries can be directed to the corresponding authors.

## Ethics Statement

The studies involving human participants were reviewed and approved by The ethical committee of Xinqiao Hospital. The patients/participants provided their written informed consent to participate in this study.

## Author Contributions

HL and YS carried out the studies, participated in data collection, statistical analysis, and the writing of the manuscript. ZY and YH contributed to the data analysis. TH, TX, and YL contributed to the data collection. JZ and JX reviewed and edited the manuscript. All authors contributed to the article and approved the submitted version.

## Funding

This study was supported by research grants from Personal Training Program for Clinical Medicine Research of Army Medical University (No. 2018XLC1007), Frontier specific projects of Xinqiao Hospital (No. 2018YQYLY004), Scientific Renovation Project of Army Medical University (No. 2019XLC3038), and Clinical Medical Research Talents Project of Army Military Medical University (No. 2019XLC3028).

## Conflict of Interest

The authors declare that the research was conducted in the absence of any commercial or financial relationships that could be construed as a potential conflict of interest.

## Publisher’s Note

All claims expressed in this article are solely those of the authors and do not necessarily represent those of their affiliated organizations, or those of the publisher, the editors and the reviewers. Any product that may be evaluated in this article, or claim that may be made by its manufacturer, is not guaranteed or endorsed by the publisher.
