# Supplementary material for: Potential Role of the Renal Arterial Resistance Index in the Differential Diagnosis of Diabetic Kidney Disease
Source: Front Endocrinol (Lausanne). 2022 Jan 14;12:731187. doi: 10.3389/fendo.2021.731187 (PMC8796316; doi:10.3389/fendo.2021.731187)
Supplement: Supplementary file 1 [file DataSheet_1.docx]

**Supplementary Table 1**.

Distribution of pathological types of non-diabetic kidney disease.

| Pathological types | Cases | Percentage (%) |
| --- | --- | --- |
| Membranous nephropathy | 38 | 27.74 |
| IgA nephropathy | 38 | 27.74 |
| Mesangial proliferative glomerulonephritis | 19 | 13.87 |
| Hypertensive nephrosclerosis | 16 | 11.68 |
| Minimal change disease | 8 | 5.84 |
| Purpura nephritis | 5 | 3.65 |
| Focal segmental glomerulonephritis. | 5 | 3.65 |
| Membranous proliferative glomerulonephritis | 3 | 2.19 |
| Lupus nephritis | 3 | 2.19 |
| Obesity-related glomerulopathy | 1 | 0.73 |
| Crescentic glomerulonephritis | 1 | 0.73 |

**Supplementary Table 2**.

Distribution of glomerular class of diabetic kidney disease.

| Pathological types | Cases | | Percentage (%) |
| --- | --- | --- | --- |
| Ⅰ | 5 | 1.51 | |
| Ⅱa | 24 | 7.23 | |
| Ⅱb | 60 | 18.07 | |
| Ⅲ | 236 | 71.08 | |
| Ⅳ | 7 | 2.11 | |

**Supplementary Table 3.**

Correlations between RI and clinical characteristics in diabetic patients with kidney disease

| Parameters | RI |  |
| --- | --- | --- |
|  | r | P value |
| Age, (years) | 0.245 | 0.000 |
| Duration of diabetes, (months) | 0.341 | 0.000 |
| SBP, (mm Hg) | 0.274 | 0.000 |
| DBP, (mm Hg) | -0.032 | 0.488 |
| BMI (kg/m^2^) | -0.143 | 0.002 |
| Hemoglobin,(g/L) | -0.424 | 0.000 |
| PLT, (10^9/L) | -0.048 | 0.303 |
| TG,(mmol/L) | -0.145 | 0.002 |
| TC,(mmol/L) | -0.042 | 0.369 |
| HbA1c,(%) | 0.033 | 0.519 |
| FBG,(mmol/L) | 0.020 | 0.660 |
| Scr,(μmol/L) | 0.335 | 0.000 |
| BUN,(mmol/L) | 0.037 | 0.000 |
| Uric acid,(μmol/L) | 0.015 | 0.752 |
| Serum albumin,(g/L) | -0.163 | 0.000 |
| Urinary protein,(g/24 h) | 0.141 | 0.003 |

RI, resistive index. SBP, systolic blood pressure; DBP, diastolic blood pressure; BMI, body mass index; PLT, platelet; TG triglyceride; TC total cholesterol; HbA1c, glycosylated hemoglobin; FBG fasting blood glucose; Scr serum creatine; BUN blood urea nitrogen; Data were presented as the mean ± standard, the median with range or counts and percentages. A two-tailed p<.0.05 was considered statistically significant.

**Supplementary Table 4**.

The equation of traditional model and RI-based model.

|  | Equation | |
| --- | --- | --- |
| Traditional model | | PDKD= exp(2.096 + 1.944Dm - 0.120BMI + 2.759DR + 0.587Gh) / [1+exp(2.096 + 1.944Dm - 0.120BMI + 2.759DR + 0.587Gh) ]. |
| RI-based model | | PDKD= exp(0.976 + 1.671Dm - 0.101BMI + 2.703DR + 0.795Gh + 1.656RI) / [1 + exp(0.976 + 1.671Dm - 0.101BMI + 2.703DR + 0.795Gh + 1.656RI)]. |

PDKD, the probability of DKD diagnosis; Dm, diabetes duration ≥ 60 months (1 yes, 0 no); BMI, body mass index; DR, diabetic retinopathy (1 yes, 0 no); Gh, HbA1c ≥ 7.0% (1 yes, 0 no); RI, Resistance index ≥ 0.66 (1 yes, 0 no).

**Supplementary Table 5**.

Previous reports on normal value of RI.

| Authors | Sample size | | Normal value of RI | | Title |
| --- | --- | --- | --- | --- | --- |
| Kim S et al. | | 28 | 0.62 ± 0.04 | Duplex sonography of the native kidney: resistive index vs serum creatinine. | |
| Platt J et al. | | 109 | 0.58 ± 0.05 | Duplex Doppler US of the kidney; differentiation of obstructive from nonobstructive dilatation. | |
| Norris C et al. | | 21 | 0.64 ± 0.05 | Non-invasive evaluation of renal artery stenosis and renovascular resistance: experimental and clinical studies. | |
| Keogan M et al. | | 58 | 0.60 ± 0.01 | Renal resistive indexes: variability in Doppler US measurement in a healthy population. | |
| Sari A et al. | | 50 | 0.56 ± 0.23 | Value of resistive index in patients with clinical diabetic nephropathy. | |
| Derchi LE et al. | | 42 | 0.64 ± 0.02 | Ultrasonographic imaging and Doppler analysis of renal changes in non-insulin-dependent diabetes mellitus. | |
| Masulli M et al. | | 37 | 0.59 ± 0.05 | Measurement of the intrarenal arterial resistance index for the identification and prediction of diabetic nephropathy. | |
| Mancini M et al. | | 73 | 0.59 ± 0.06 | Renal duplex sonographic evaluation of type 2 diabetic patients. | |

Data were presented as the mean ± standard.
